# Persistent, Mobile, Toxic: The Effects of Chemical Warning Labels on Public Risk Perception

**DOI:** 10.1021/acs.estlett.5c01231

**Published:** 2026-03-09

**Authors:** Ellise Suffill, Nina Vaupotič, Jule Schlösser, Sarah E. Hale, Mathew P. White, Sabine Pahl

**Affiliations:** † Environmental Psychology Group, University of Vienna, Vienna 1010, Austria; ‡ Cognitive Science Hub, University of Vienna, Vienna 1090, Austria; § Department of Water Supply, DVGW-Technologiezentrum Wasser, D-76139 Karlsruhe, Germany; ∥ Environment and Climate Hub, University of Vienna, Vienna 1090, Austria

**Keywords:** PFAS, PM substances, product labels, risk perception, risk communication

## Abstract

Persistent, mobile, and toxic (PMT) chemicals, such as per- and polyfluoroalkyl substances (PFAS), pose significant environmental and health risks. Public risk perception is an important driver of change in regulation and consumption patterns. Previous research has investigated the effect of toxicity information, but little is known about public perception of chemical properties such as persistence or mobility alone or in combination with toxicity. In this study, 328 participants rated one of two everyday products in terms of affect, concern, and policy support. Labels indicated the presence of persistent (P), mobile (M), and toxic (T) chemicals either in isolation or combined (PM, PT, MT, and PMT), compared to a control with no label. When labels indicated only a single chemical property, toxicity elicited the strongest risk response, and this was amplified for products with high body contact (dental care) compared to low body contact (household cleaner). Labels warning of all three properties together (PMT) triggered the strongest risk response while demonstrating a nonadditive relationship. These findings suggest that the public is sensitive to warnings about different chemical properties beyond toxicity. Informing the public of diverse chemical properties has the potential to change behavior and encourage support for stricter regulation.

## Graphical abstract

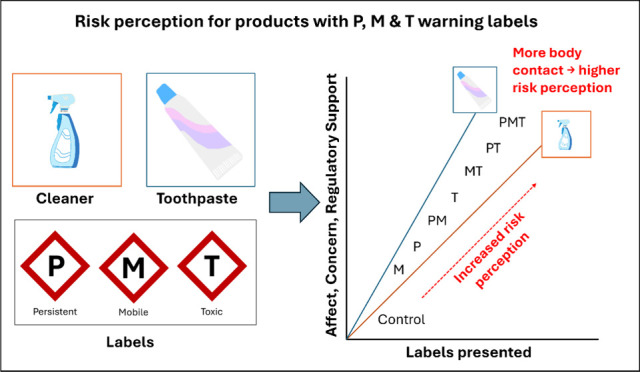

## 1 Introduction

Persistent, mobile, and toxic (PMT) substances, like per- and polyfluoroalkyl substances (PFAS), are used in everyday products such as stain- and water-resistant clothing, cookware, and food packaging.^1^ However, PFAS are linked to health risks (e.g., cancer and developmental issues)^2–5^ and environmental harm.^6–8^ Their mobility means they can travel large distances with water, and their persistence increases long-term exposure. Cousins et al.^9^ suggested society has exceeded safe PFAS levels, requiring urgent action from all stakeholders. A universal PFAS restriction^10^ was first proposed in 2023 as a solution to PFAS pollution. As of early 2026, the proposal remains under review. In the absence of binding restrictions, PMT labels can raise awareness and support an informed consumer choice. If restrictions are adopted, labels may become less relevant, though they remain useful for explaining product changes, facilitating trade-off acceptance, and maintaining trust among consumers, industry, and regulators.^11^

While not explicitly referenced in the restriction proposal, the concept of essential use (EUC) is another pathway to reduce the use of harmful chemicals. The EUC proposes to allow uses only when these are strictly necessary and when suitable alternatives are unavailable.^12^ Such essential considerations are not fixed. They may evolve with societal needs, awareness, and technological change, and labels could shift stakeholder views on what counts as essential at any given point in time.^13,14^

Pretesting labels can further clarify consumer interpretation and behavioral responses and help shape industry and policy decisions,^15–18^ informing measures like classification, labeling, and packaging regulation^19^ and the EU digital product passport.^20^ However, research on communicating about PFAS and PMT substances is limited.^14^

### 1.1 Factors Influencing Public Risk Perception of PMT Substances

Risk perception refers to subjective attitudes about risks and benefits, e.g., of products or substances.^21–23^ Unlike experts, who typically rely on technical analyses, the public, i.e., nonexperts, incorporate social, cultural, and psychological factors, such as voluntariness (e.g., whether exposure is a choice) and equity (e.g., who is most affected),^24,25^ into their judgment. Psychology has conceptualized attitudes as having affective (emotional responses), cognitive (judgements and beliefs), and behavioral (intended or actual actions) components.^26^ Applied to environmental risks, affect reflects positive or negative feelings,^27^ cognition reflects judgements of severity and likelihood,^28^ and behavior reflects actions to manage or reduce exposure.^29,30^

In the context of risk perception of chemicals, “intuitive toxicology” describes intuitions about the risk of substances, for example, the assumption that any exposure to toxic chemicals is harmful, without fully appreciating dosage.^31^ Negative perception of chemicals may also be driven by so-called “heuristics”, mental shortcuts that save cognitive capacity.^32^ Thus, a toxicity label may trigger immediate negative emotions, which then drives down benefit perceptions of the product, akin to an “affect heuristic”.^25,33–37^ The public also exhibit heightened concern about synthetic chemicals in particular, perceiving them as more dangerous than natural chemicals,^36,38^ in line with a “nature is better” heuristic.^39^ When combined, these heuristics may add up to a general “chemophobia” in the public.^33^

Other research suggests that when faced with multiple simultaneous risks, people may focus on a single cue over others,^40^ average risks across cues,^41^ or underestimate their cumulative impact.^42^ While not specific to chemical risk, these findings highlight the range of nonexpert responses to cumulative hazards and seem relevant for public perceptions of different kinds of chemical properties such as PMT.

Apart from characteristics of hazards, characteristics of the perceiver such as direct experience with a hazard, knowledge and trust in authorities, and socio-demographic factors are also associated with risk perception. First, direct contact with chemicals and greater personal relevance increases risk perception, boosts regulatory support, and affects consumer choices.^43,44^ For instance, PFAS in personal care products like makeup triggers higher concern than PFAS in water or soil.^45^ Second, the public rely on trust in science and regulation to assess chemical risks as they lack specialized knowledge about risk assessment.^46^ Studies show only weak positive links between knowledge and risk perception in areas like COVID-19,^47^ climate change,^30^ and chemicals generally,^36^ suggesting that knowledge plays a role but is not the main factor related to risk perception. Lastly, socio-demographic factors are related to risk perception. Women and older adults generally exhibit higher risk perception across domains, including chemicals.^30,31^ For PFAS, in particular, women report greater concern and lower tolerance for these substances in products than do men.^48^

Finally, contextual factors such as labeling can also affect risk perception toward chemicals in products. Eco-labels such as “GMO-free” increase willingness to pay for products perceived as healthier or safer.^44,49,50^ However, labels can also lead to misinterpretations: cleaning products labeled as eco-friendly are viewed as safer, even when risks are present.^43,51^ Warning labels, mandated for some cleaning agents,^43,52^ communicate risks through pictograms, signal words (e.g., “danger”), and hazard statements. While sometimes effective, their impact varies across cultures.^53–55^ Labels highlighting health and environmental risks of PFAS in outdoor clothing were found to increase willingness to pay for PFAS-free alternatives.^17^ To date, no research has examined how labels warning of combinations of persistence, mobility, and toxicity affect public risk perception of everyday products.

### 1.2 Current Study

An online experiment examined public risk perceptions of products containing PMT chemicals. Participants rated labeled and unlabeled versions of two fictitious products (toothpaste and household cleaner) for affect, concern, and regulatory importance, and the study tested whether body contact influenced these perceptions.

For H1, toxicity will have a greater impact on risk perceptions than persistence or mobility.

For H2, the higher-contact product (toothpaste) will elicit more negative risk perceptions than the lower-contact product (cleaner), particularly when combined with PMT labels.

## 2 Materials and Methods

The study was approved by the University of Vienna ethics committee (no. 01043), followed STROBE guidelines (v4), and adhered to Open Science principles, including preregistration and an a priori power analysis (https://osf.io/zvgds).

### 2.1 Participants

A total of 328 participants (160 males, 165 females, 3 other; mean age of 30.67, SD of 11.27) were tested in an online study. Participants were recruited within Europe via Prolific (app.prolific.com) and required to be more than 18 years of age as well as fluent in English. The experiment was run on Qualtrics and took a median of 9:03 min, and participants were paid £1.50/€1.70. Full demographics are listed in Table S1.

### 2.2 Materials

Two sets of stylized images were created for the household cleaner and toothpaste, accompanied by a short description of the PMT properties (Table S2), resulting in eight versions: control, P, M, T, PM, PT, MT, and PMT in combination (Figure 1). Labels were generated for this study and not official EU warnings. To validate our assumption regarding perceived body contact of products, we ran an independent *t* test on participants’ ratings of body contact during product use. Toothpaste (*M* = 5.46) was rated higher than cleaner, as expected (*M* = 3.87) (*t*(327) = −9.36; *p* < 0.001). Participants also received a short introduction to chemicals and EU regulation (Table S3).

**Figure 1:**
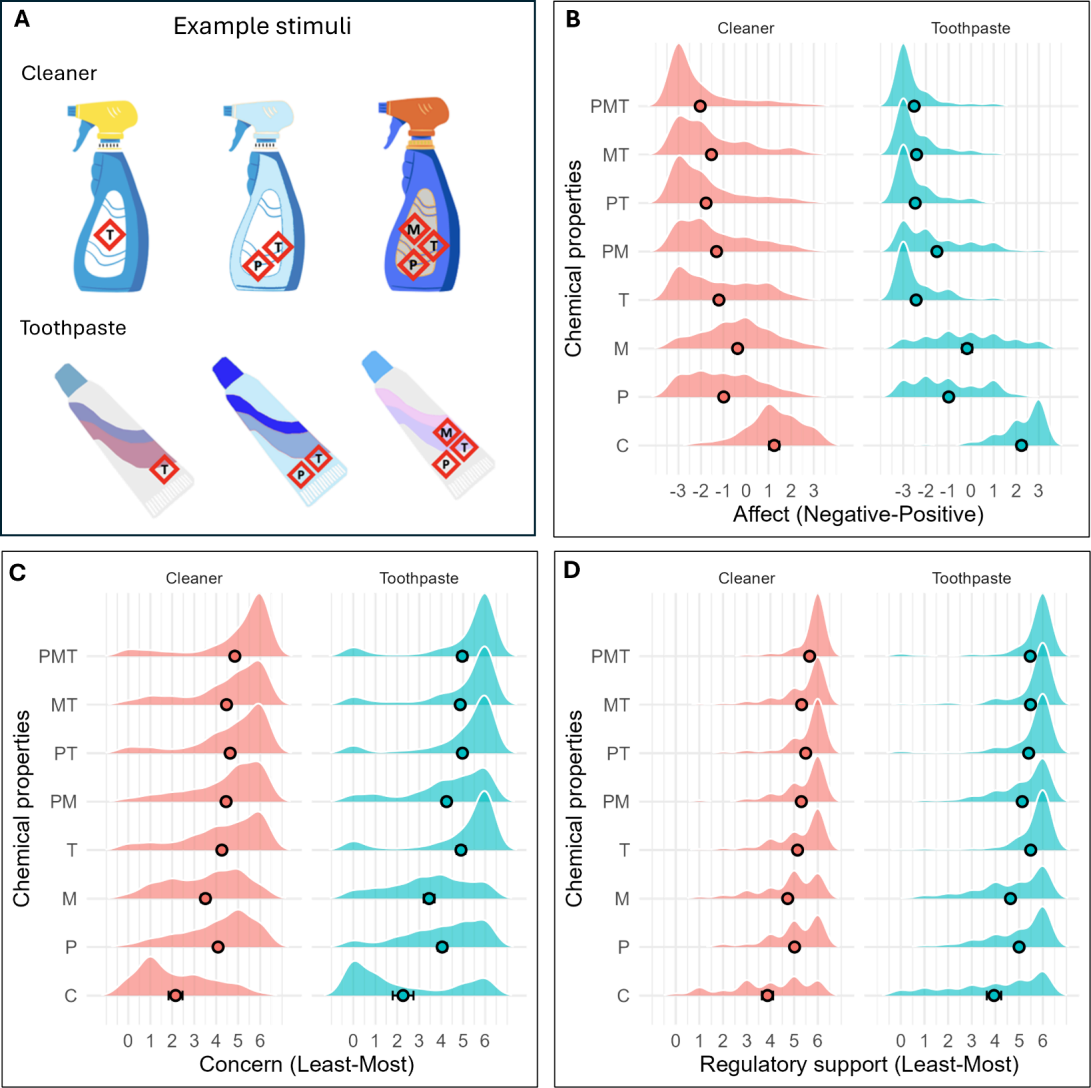
(A) Example stimuli and (B) scores for affect, (C) concern, and (D) importance of regulation are shown by label and product. Points represent group averages; density reflects the response frequency, and bars indicate 95% confidence intervals (CIs; note that these are small and visible under only some control conditions). Legend: C, control; P, persistent; M, mobile; T, toxic.

### 2.3 Ratings

The main risk perception indices were single-item measures. Affect: “I see this product as something that is ...” (1 = very negative, 7 = very positive).^56^ Concern: “How concerned are you personally with using this product in your daily life?” (1 = not concerned at all, 7 = very concerned).^30^ Support for regulation: “I think it is important to regulate the use of this product” (1 = completely disagree, 7 = completely agree). These were intended to capture the affective (affect), cognitive (concern), and behavioral (support for regulation) components of risk perception.^29,30^ Participants also reported their trust in labels and regulatory authorities and their prior knowledge of PMT chemicals and PFAS (Table S4).

### 2.4 Procedure

After providing consent and basic information about EU chemical regulation, participants rated their general trust in labels and regulatory authorities. Participants were then randomly assigned to view either the household cleaner (*N* = 162) or the toothpaste (*N* = 166) and rated affect, concern, and importance of regulation for each product. The control was always rated first, followed by P, M, T, PM, MT, PT, and PMT labels in random order. Finally, participants reported prior PFAS knowledge, completed a definitions check, and provided socio-demographic data.

### 2.5 Analytic Approach

Data analysis was carried out in R,^57^ using the “lme4” package^58^ for linear mixed-effects regression (LMM) with by-subject random intercepts and inspecting confidence intervals to interpret differences. LMMs include fixed effects (i.e., type of label and product), which estimate the influence of predictors of interest in the outcome (i.e., affect, concern, and regulatory support), and random effects, which account for additional sources of variability, such as differences between participants. Affect scores were coded from −3 (very negative) to 3 (very positive). Concern was coded from 0 (not at all concerned) to 6 (very concerned), and support for regulation was coded from 0 (completely disagree) to 6 (completely agree) for easier interpretability. Chemical properties (control vs persistence, mobility, toxicity) were coded as binary variables (e.g., 1 = toxic, 0 = not toxic). An additional model added trust in labeling and regulation, PFAS knowledge, age, and gender as covariates (Table S7).

## 3 Results

### 3.1 Descriptives

Figure 1 visualizes scores by conditions (presented in detail in Table S5). Associations among risk perceptions, trust, PFAS knowledge, and demographics (see Table S6) were moderate to weak (all rs < 0.20). Correlations between the different elements of risk perceptions were as follows: *r* = −0.46 for affect–concern, *r* = −0.47 for affect–regulation support, and *r* = 0.45 for concern–regulation support.

### 3.2 Models Testing Risk Perceptions toward Chemicals across Products

Positive estimates (*B*) indicate higher affect, concern, or perceived regulatory importance, while negative estimates indicate lower levels; model baselines reflect outcomes for control products with no label.

#### 3.2.1 Affect

Affect measured participants’ positive or negative feelings toward each product. The control intercept was slightly positive (*B* = 1.25; *t* = 9.96; *p* < 0.001). Adding any single chemical property shifted affect from positive to negative, with toxicity having the strongest effect. Combinations of two or more properties also produced a negative effect, but the effects were not strictly additive (Figure 1B).

##### 3.2.1.1 Effects of Single Properties

Any single chemical property label led to a more negative effect compared to the control (no label). Toxicity had the strongest effect (for the cleaner, *B* = −2.44, *t* = −20.30, and *p* < 0.001; for the toothpaste, *B* = −4.68, *t* = −41.07, and *p* < 0.001). Persistence (for the cleaner, *B* = −2.23, *t* = −18.52, and *p* < 0.001; for the toothpaste, *B* = −3.23, *t* = −28.31, and *p* < 0.001) and mobility (for the cleaner, *B* = −1.61, *t* = −13.38, and *p* < 0.001; for the toothpaste, *B* = −2.40, *t* = −21.02, and *p* < 0.001) also triggered a more negative affect than the control. Confidence intervals (CIs) demonstrated some differences between properties. For the cleaner, toxicity (95% CIs: −2.68, −2.21) was not perceived as more negatively than persistence (−2.46, −1.99; i.e., overlapping CIs), though both were perceived more negatively than mobility (−1.85, −1.37). For the toothpaste, effects were similar, but toxicity (−4.91, −4.46) was perceived more negatively than persistence (−3.45, −3.00) or mobility (−2.62, −2.17).

##### 3.2.1.2 Two-Way Interactions

Here, the positive estimates suggest that the combined effect of two properties on the effect is less negative than the sum of their individual effects: persistence × mobility (for the cleaner, *B* = 1.30, *t* = 7.65, and *p* < 0.001; for the toothpaste, *B* = 1.85, *t* = 11.48, and *p* < 0.001), persistence × toxicity (for the cleaner, *B* = 1.66, *t* = 9.78, and *p* < 0.001; for the toothpaste, *B* = 3.19, *t* = 19.82, and *p* < 0.001), and toxicity × mobility (for the cleaner, *B* = 1.29, *t* = 7.57, and *p* < 0.001; for the toothpaste, *B* = 2.43, *t* = 15.07, and *p* < 0.001).

##### 3.2.1.3 Three-Way Interaction

The negative effect suggests that adding a third property led to a steeper decline in affect than would be expected from the sum of the effects of single chemical properties, with PMT producing the most negative affect (e.g., for the cleaner, *M* = −2.03, SD = 0.92): persistence × mobility × toxicity (for the cleaner, *B* = −1.25, *t* = −5.19, and *p* < 0.001; for the toothpaste, *B* = −1.92, *t* = −8.40, and *p* < 0.001).

#### 3.2.2 Concern

This item indicates participants’ personal concern about using each product. The control intercept was relatively low (*B* = 2.13; *t* = 15.40; *p* < 0.001). As with affect (Figure 1C), single properties, especially toxicity, increased concern, two-way combinations produced greater but less-than-additive effects, and the full three-property combination elicited the largest increase.

##### 3.2.2.1 Effects of Single Properties

Toxicity produced the most concern (for the cleaner, *B* = 2.15, *t* = 15.02, and *p* < 0.001; for the toothpaste, *B* = 2.66, *t* = 14.89, and *p* < 0.001), followed by persistence (for the cleaner, *B* = 1.97, *t* = 13.71, and *p* < 0.001; for the toothpaste, *B* = 1.80, *t* = 10.06, and *p* < 0.001) and mobility (for the cleaner, *B* = 1.40, *t* = 9.78, and *p* < 0.001; for the toothpaste, *B* = 1.18, *t* = 6.61, and *p* < 0.001). According to the confidence intervals, for the cleaner, toxicity (95% CIs: 1.87, 2.44) was perceived more negatively than mobility (1.12, 1.68), while persistence (1.69, 2.25) and toxicity had similar effects. For the toothpaste, toxicity (2.31, 3.01) produced greater concern than persistence (1.45, 2.15) and mobility (0.83, 1.53).

##### 3.2.2.2 Two-Way Interactions

Negative estimates show that concern increased less than expected when the two properties were combined: persistence × mobility (for the cleaner, *B* = −1.06, *t* = −5.23, and *p* < 0.001; for the toothpaste, *B* = −0.97, *t* = −3.83, and *p* < 0.001), persistence × toxicity (for the cleaner, *B* = −1.58, *t* = −7.78, and *p* < 0.001; for the toothpaste, *B* = −1.74, *t* = −6.88, and *p* < 0.001), and toxicity × mobility (for the cleaner, *B* = −1.19, *t* = −5.86, and *p* < 0.001; for the toothpaste, *B* = −1.21, *t* = −4.40, and *p* < 0.001).

##### 3.2.2.3 Three-Way Interaction

Adding a third property led to a stronger increase in concern than would be expected from simply summing the single properties: persistence × mobility × toxicity (for the cleaner, *B* = 1.05, *t* = 3.67, and *p* < 0.001; for the toothpaste, *B* = 1.00, *t* = 2.80, and *p* = 0.01).

#### 3.2.3 Importance of Regulation

This item indicated participants’ support for regulating each product. The control intercept was already relatively high (*B* = 3.87; *t* = 39.57; *p* < 0.001). Consistent with affect and concern, single properties, especially toxicity, increased support, two-way combinations produced less-than-additive effects, and all three properties together elicited the largest increase.

##### 3.2.3.1 Effects of Single Properties

Toxicity had the strongest effect (for the cleaner, *B* = 1.28, *t* = 12.59, and *p* < 0.001; for the toothpaste, *B* = 1.54, *t* = 13.23, and *p* < 0.001), followed by persistence (for the cleaner, *B* = 1.15, *t* = 11.34, and *p* < 0.001; for the toothpaste, *B* = 1.06, *t* = 9.10, and *p* < 0.001) and mobility (for the cleaner, *B* = 0.85, *t* = 8.37, and *p* < 0.001; for the toothpaste, *B* = 0.66, *t* = 5.69, and *p* < 0.001). Confidence intervals showed overlap between properties, suggesting similar effects on the regulatory importance for both products.

##### 3.2.3.2 Two-Way Interactions

Negative estimates suggest that the regulatory importance increased less than expected if two properties were simply additive: persistence × mobility (for the cleaner, *B* = −0.58, *t* = −4.06, and *p* < 0.001; for the toothpaste, *B* = −0.53, *t* = −3.24, and *p* = 0.001), persistence × toxicity (for the cleaner, *B* = −0.81, *t* = −5.64, and *p* < 0.001; for the toothpaste, *B* = −1.14, *t* = −6.95, and *p* < 0.001), and toxicity × mobility (for the cleaner, *B* = −0.69, *t* = −4.80, and *p* < 0.001; for the toothpaste, *B* = −0.68, *t* = −4.11, and *p* < 0.001).

##### 3.2.3.3 Three-Way Interaction

Adding a third property led to a sharper increase in regulatory importance than would be expected from the sum of the single property effects: persistence × mobility × toxicity (for the cleaner, *B* = 0.58, *t* = 2.83, and *p* = 0.005; for the toothpaste, *B* = 0.61, *t* = 2.62, and *p* = 0.01).

### 3.3 Differences by Product and Covariates

Table S7 reports a model with both products combined and additional covariates. The addition of these covariates did not alter the main pattern of the results.

#### 3.3.1 Product Differences

For affect, there was an interaction between the combined PMT label and product (*B* = −0.67; *t* = −2.01; *p* < 0.001), with PMT leading to a more negative effect for the toothpaste than for the cleaner. For concern, there was a product × toxicity interaction (*B* = 0.51; *t* = 2.21; *p* = 0.03), with a steeper increase in concern for the toothpaste. No product differences were found for regulatory importance (*p* > 0.05).

#### 3.3.2 Covariates

Due to the nonrepresentative sample, covariate associations should not be generalized. Covariates were included only to reduce residual confounding of label and product effects; effects are reported below for the sake of completeness. Greater trust in regulation was associated with greater concern (*B* = 0.12; *t* = 2.03; *p* = 0.04) and regulatory importance (*B* = 0.18; *t* = 4.11; *p* < 0.001). Prior knowledge of PFAS was associated with higher regulatory importance (*B* = 0.09; *t* = 2.46; *p* = 0.01). Female participants reported more negative affect (*B* = 0.46; *t* = 4.02; *p* < 0.001) and greater regulatory importance (*B* = −0.26; *t* = −2.48; *p* = 0.01) than did males. Older participants reported more negative affect (*B* = −0.01; *t* = −2.36; *p* = 0.02), greater concern (*B* = 0.02; *t* = 3.83; *p* < 0.001), and greater regulatory importance (*B* = 0.02; *t* = 3.57; *p* < 0.001).

## 4 Discussion

This study examined public risk perceptions, affect, concern, and support for regulation, of PMT substances in everyday products. Participants viewed stylized images of toothpaste and household cleaner with labels highlighting persistence, mobility, and toxicity (alone or combined) or a no-label control. Risk perceptions varied by property and product with toxicity eliciting the strongest responses. Persistence and mobility also raised concern, and participants generally supported regulation of chemicals in the products.

Toxicity was hypothesized to influence risk perceptions the most, due to greater public familiarity with the term potentially eliciting an affect heuristic.^33–36,59^ Consistent with this, toxicity triggered more negative effects and concerns than did persistence or mobility. This effect was amplified for the product with more direct body contact, i.e., toothpaste. Persistence also caused more negative affect than mobility, warranting further research on how nonexperts understand such properties and beliefs about how they affect the environment and human health. These findings are important to consider in relation to current expert advice to prioritize persistent and mobile substances, even if nontoxic (e.g., under REACH^60^).

While combining two properties (e.g., PM) increased risk perception compared to a single property, this effect was not simply additive. A significant persistence × mobility × toxicity interaction indicated that two-property combinations had less-than-additive effects on negative effects, whereas the full three-property combination elicited the strongest response. This suggests that PMT labels are perceived as a distinct high-hazard category rather than an additive sum, consistent with prior evidence that nonexperts integrate multiple risks nonadditively^41,42^ and extending cue-dominance research^40^ to the chemical domain by showing that toxicity dominates until a PMT threshold is reached.

Perceived body contact partially influenced the risk perceptions. Toothpaste was rated as having higher body contact than cleaner, and the combined PMT label caused more negative affect for toothpaste. This supports findings that physical contact or ingestion heightens feelings of risk.^43^ However, concern and regulatory importance were similar across the products.

We observed differences in the sensitivity of outcome measures to chemical property information. Affect was the most responsive, shifting strongly in response to single properties, especially toxicity, and for products with greater perceived body contact. Concern showed a similar pattern, increasing reliably with additional properties but with smaller overall changes. In contrast, support for regulation was relatively stable and high across properties, perhaps indicative of a ceiling effect that reflects generally strong support for the regulation of such hazardous substances. Together, these findings suggest that chemical property information primarily shapes immediate emotional responses, while regulatory preferences are more robust to informational nuance.^37^ While we analyzed our outcome measures separately, future work could examine how these measures relate to each other (e.g., in parallel or serially) and the extent to which they add explanatory power for predicting downstream outcomes such as behavioral intentions or policy support. In addition, distinguishing between concern for human health versus environmental impacts may also reveal differential responses to PMT labels.^61–63^

Practically, labeling products with PMT properties may increase risk perception and subsequently reduce consumption and promote sustainable choices at the same time as increasing support for management of these substances.^50,64,65^ Highlighting multiple chemical properties might be the most effective way to influence public perception and behavior. However, the relationship is complex and nonlinear, with the product’s proximity to the human body (e.g., toothpaste vs cleaner) amplifying some label effects.^43^ These findings could guide label design and field trials to study how concerns influence real-world purchasing behavior and support for policies to reduce PFAS use. Whether such patterns hold for combinations of other chemical properties, or when undesirable properties are mixed with desirable properties, requires further research.

### 4.1 Limitations and Future Directions

This study focused on two product exemplars, which limits generalizability. Future research should examine a broader range of products to determine whether PMT labeling effects extend across domains and to identify contexts in which PMT chemicals are nonessential (e.g., if a PMT warning reduces sales, the substance may be nonessential in this use). Also, single-item indices were used to reduce the participant burden in a multi-item test scenario; future studies could incorporate validated multi-item scales where feasible.

Our sample was not nationally representative in terms of age, income, or education, so caution is needed in generalizing these findings. While not the focus of this research, replication in more representative samples is recommended to examine how demographic factors relate to chemical risk perception. For example, chemical education level might be important in explaining differences in the general “chemophobia” often reported by the public.^36^

Finally, a common critique of product labeling is that it shifts responsibility onto consumers while industry practices remain unchanged (cf. tobacco and alcohol^66–68^ ), and this may also apply to PMT-containing products. However, labels may also empower consumers by increassing awareness and support for stricter regulation while preserving choice. Future research should examine how labels shape responsibility perceptions, intentions, and policy preferences and how they function alongside broader risk communication strategies such as public education, standardized reporting, and guidance on safer alternatives.

### 4.2 Implications

The results indicate that chemical labels on everyday products influence public risk perceptions, increasing negative affect, concern, and support for regulation, with toxicity having the strongest effect of any single property. Combined PMT labels produced the strongest risk response, but the relationship between risk perception and the number of chemical properties reported was complex and nonlinear. Greater perceived body contact heightened risk responses. Citizen awareness and consumer choice are two of several pathways to reducing hazardous substances and should be considered alongside legal and economic drivers to support the phase-out of hazardous substances such as PFAS.

## Supplementary Material



## Notes

Additional participant sample demographics (Table S1), descriptions associated with each chemical property (Table S2), study introduction for participants regarding chemicals and EU regulation (Table S3), full phrasing of questions to participants (Table S4), means and SDs of risk perception outcomes by chemical properties (Table S5), correlations among risk perception outcomes, demographics, trust, and prior knowledge (Table S6), and model for affect, concern, and regulatory support across both products with covariates included (Table S7) (PDF)
