# Supplementary material for: Persistent, Mobile, Toxic: The Effects of Chemical Warning Labels on Public Risk Perception
Source: Environ Sci Technol Lett. 2026 Mar 9;13(4):459–66. doi: 10.1021/acs.estlett.5c01231 (PMC13085796; doi:10.1021/acs.estlett.5c01231)
Supplement: Supplementary file 1 [file ez5c01231_si_001.pdf]

Persistent, Mobile, Toxic: The Effects of Chemical Warning Labels on Public Risk Perception

Ellise Suffill[1*], Nina Vaupotič[1,4], Jule Schlösser[1], Sarah E. Hale[3], Mathew P. White[1,2,4], Sabine Pahl[1,4.]

1 Environmental Psychology Group, University of Vienna, Vienna, 1010, Austria

2 Cognitive Science Hub, University of Vienna, Vienna, 1090, Austria

3 Department of Water Supply, DVGW-Technologiezentrum Wasser, D-76139, Karlsruhe, Germany

4 Environment and Climate Hub, University of Vienna, Vienna, 1090, Austria

* Denotes corresponding author. Email: *ellise.suffill@univie.ac.at*

Supporting Information

Table S1.

Additional demographic information regarding participants (averages and frequencies)

**Average age (SD)**	30.73 (11.26); range = 18-70 years
**Gender**	Male = 158<br>Female = 163
**Education**	Did not complete high school/secondary education = 7<br>Completed secondary school certificate = 64<br>Completed Baccalaureate/A-levels (school attendance up to age 18) = 66<br>Completed a university degree = 184
**Political orientation**<br>**(0 = left-wing - 10 = right-wing)**	0 = 16; 1 = 12; 2 = 48; 3 = 55; 4 = 55; 5 = 36; 6 = 33; 7 = 25; 8 = 17; 9 = 1; 10 = 4; NA = 19
**Income**	Live comfortably = 100<br>Get along = 142<br>Get along with difficulty = 67<br>Get along with high difficulty = 12

Table S2.

Descriptions provided for each chemical characteristic.

Persistent	*Persistent (P) means that the chemicals will stay in the environment and living organisms, so that concentrations of the chemical increase over time.*
Mobile	*Mobile (M) means that the chemicals can be transported by water, so that they may reach regions where they have never been produced or used.*
Toxic	*Toxic (T) means that the chemicals can cause harm to exposed humans and environment.*

Table S3.
Introduction to chemicals and regulation shown to participants at the start of the study.

"Chemicals and their regulation in the EU. *Firstly, we'll tell you a little bit about how chemicals and their risks are managed within the EU. Please read the following information carefully, as it is important to completing the survey.* *Chemicals serve many useful functions in both industry and everyday life, but they come with potential positive and negative impacts. For example:* ● *Chemicals can make products more durable, so that they last longer, or make surfaces easier to clean.* ● *However, because of their structure, chemicals can also escape into the environment and pose risks to both human health and the natural environment.*
What happens when a chemical risk is deemed to be high? *In this case, authorities - like the European Commission - may decide to regulate a chemical use, meaning the chemical may be banned completely or that it is only allowed to be used in the 'most essential' cases.* *This can have both positive and negative knock-on effects:* ● *A positive effect is that regulation can be used to reduce the chemical risk to health and the environment, by banning or reducing the production and use of a risky chemical.* ● *A negative effect is that industry may have to find new ways to produce products without the chemical (e.g., by finding chemical alternatives) and this may lead to the product performing less well or being more expensive for consumers to buy.*

Table S4.

Risk attitude indices.

Measure	Phrasing	Additional information
Affect	"I see this product as something that is…" (1 = very negative, 7 = very positive)[1];	These measures reflected emotional (affect), cognitive (concern) and behavioural (support for regulation) responses[2]. Single-item indices were used to minimize time and cognitive load.
Concern	"How concerned are you personally with using this product in your daily life?" (1 = not concerned at all, 7 = very concerned) [3]	
Support for regulation	"I think it is important to regulate the use of this product" (1 = completely disagree, 7 = completely agree).	
Trust	"When I come across labels on products indicating different characteristics, I trust their authenticity" and "I trust the EU government to lead an effective regulation strategy for different chemicals." Responses ranged from strongly disagree (1) to strongly agree (7).	Similar items have been used in previous studies[4,5].
Prior knowlegde	"Please rate how much you knew about each of the topics below, before taking part in this survey" for "Persistent, Mobile and Toxic chemicals" and "PFAS/Forever chemical" Responses ranged from no knowledge at all (1) to a lot of knowledge (7)."	Questions were asked post-experiment to avoid priming and were adapted from other subjective knowledge scales[6].

1. Peters E, Slovic P. Affective asynchrony and the measurement of the affective attitude component. Cogn Emot. 2007;21(2):300–29.

2. Aronson E, Wilson TD, Akert RM, Sommers SR. Social psychology. ninth. England: Pearson; 2016.

3. Linden S. The social-psychological determinants of climate change risk perceptions: Towards a comprehensive model. J Environ Psychol. 2015;41:112–24.

4. Rupprecht CD, Fujiyoshi L, McGreevy SR, Tayasu I. Trust me? Consumer trust in expert information on food product labels. Food Chem Toxicol. 2020;137:111170.

5. Gorton M, Tocco B, Yeh CH, Hartmann M. What determines consumers' use of eco-labels? Taking a close look at label trust. Ecol Econ. 2021;189:107173.

6. Goebel M, Wardropper CB. Trust and subjective knowledge influence perceived risk of lead exposure. Risk Anal. 2024;44(5):1204–18.

Table S5.

Means and SDs for each of the outcome variables by chemical characteristic(s). P = Persistent, M = Mobile, T = Toxic, as represented on product label.

	Cleaner						Toothpaste					
**Chemical characteristic(s)**	**Affect** *M (SD)*		**Concern** *M (SD)*		**Support for regulation** *M (SD)*		**Affect** *M (SD)*		**Concern** *M (SD)*		**Support for regulation** *M (SD)*	
Control (no label)	1.25	(-1.41)	2.15	(2.01)	3.87	(1.49)	2.24	(1.26)	2.26	(2.97)	3.94	(1.92)
P	-0.99	(0.89)	4.09	(1.00)	5.02	(0.74)	-0.99	(1.13)	4.05	(1.28)	5.00	(0.77)
M	-0.37	(1.05)	3.51	(1.22)	4.73	(0.88)	-0.17	(1.39)	3.45	(1.56)	4.64	(1.00)
T	-1.20	(1.10)	4.26	(1.07)	5.15	(0.80)	-2.43	(0.70)	4.90	(1.24)	5.49	(0.73)
PM	-1.30	(1.02)	4.45	(1.08)	5.30	(0.77)	-1.51	(1.05)	4.25	(1.13)	5.13	(0.65)
PT	-1.77	(0.87)	4.64	(1.15)	5.49	(0.71)	-2.47	(0.69)	4.97	(1.07)	5.40	(0.82)
MT	-1.53	(0.96)	4.47	(1.10)	5.32	(0.63)	-2.41	(0.65)	4.87	(1.12)	5.48	(0.72)
PMT	-2.03	(0.92)	4.85	(1.12)	5.66	(0.71)	-2.52	(0.79)	4.96	(1.27)	5.47	(0.94)

Note. Affect coded as: -3-3; Very negative-Very positive. Concern: 0-6; Not at all concerned-Very concerned. Support for regulation: 0-6; Completely Disagree-Completely agree.

Table S6.

Pearson's correlations between outcomes and demographics variables, trust and prior knowledge averaged by participant.

	Trust labels	Trust reg	PMT knowledge	PFAS/ forever chems knowledge	Age	Education	Income	Politics	Affect	Concern
**Trust labels**	1									
**Trust reg**	0.55	1								
**PMT knowledge**	-0.14	-0.08	1							
**PFAS/ forever chems knowledge**	-0.11	-0.14	0.66	1						
**Age**	0	-0.01	-0.09	-0.08	1					
**Education**	0.05	0.04	0.04	0.07	0.06	1				
**Income**	-0.08	-0.04	0.01	0.03	0	-0.06	1			
**Politics**	-0.04	-0.17	0.06	0.01	0.11	-0.01	-0.08	1		
**Affect**	0.03	0.02	0.02	-0.02	-0.1	-0.05	0.03	0.08	1	
**Concern**	0.03	0.07	0.02	0.03	0.14	0.08	0.01	-0.07	-0.46	1
**Support for regulation**	0.1	0.15	0.06	0.04	0.14	0.15	0.01	-0.13	-0.47	0.45

As gender is presented as a binary variable, we additionally used point-biserial tests to check for associations between gender and the three outcome variables (gender was coded as female = 0; male = 1):

Affect and gender, $r = 0.13$ ($t(2414) = 6.62$, $p < .001$).

Concern and gender, $r = -0.05$ ($t(2414) = -2.38$, $p = .02$).

Support for regulation and gender, $r = -0.13$ ($t(2414) = -6.30$, $p < .00$

Table S7.

Outcomes across chemical characteristics for affect, concern and support for regulation with covariates included in the models. C = Control; P = Persistent, M = Mobile, T = Toxic, as represented on label. Effects significant at minimum p > .05 marked in bold.

	Affect				Concern				Support for regulation			
Predictors	*B*	*CI*	*t*	*p*	*B*	*CI*	*t*	*p*	*B*	*CI*	*t*	*p*
Intercept (C)	**1.06**	0.33, 1.80	2.84	**.01**	0.61	-0.28, 1.49	1.34	.18	**2.30**	1.64, 2.96	6.83	**<.001**
P	**-2.23**	-2.46, -2.00	-18.87	**<.001**	**1.97**	1.64, 2.29	11.99	**<.001**	**1.15**	0.94, 1.37	10.45	**<.001**
M	**-1.61**	-1.84, -1.38	-13.64	**<.001**	**1.40**	1.08, 1.72	8.55	**<.001**	**0.85**	0.64, 1.07	7.72	**<.001**
T	**-2.44**	-2.67, -2.21	-20.69	**<.001**	**2.15**	1.83, 2.48	13.13	**<.001**	**1.28**	1.07, 1.50	11.60	**<.001**
Product (cleaner vs. toothpaste)	**1.05**	0.74, 1.36	6.59	**<.001**	0.06	-0.34, 0.46	0.30	.76	0.07	-0.22, 0.35	0.47	.64
Age	**-0.01**	-0.02, -0.00	-2.36	**.02**	**0.02**	0.01, 0.04	3.83	**<.001**	**0.02**	0.01, 0.03	3.57	**<.001**
Gender (Female vs. male)	**0.46**	0.24, 0.69	4.02	**<.001**	-0.12	-0.39, 0.15	-0.87	.38	**-0.26**	-0.46, -0.05	-2.48	**.01**

Avg trust in regulation	0.06	-0.03, 0.16	1.29	.20	**0.12**	0.00, 0.24	2.03	**.04**	**0.18**	0.09, 0.27	4.11	**<.001**
Prior knowledge of PFAS	-0.01	-0.09, 0.07	-0.24	.81	0.08	-0.01, 0.18	1.75	.08	**0.09**	0.02, 0.16	2.46	**.01**
P×M	**1.30**	0.97, 1.63	7.80	**<.001**	**-1.06**	-1.52, -0.61	-4.57	**<.001**	**-0.58**	-0.89, -0.28	-3.74	**<.001**
P×T	**1.66**	1.34, 1.99	9.97	**<.001**	**-1.58**	-2.03, -1.12	-6.80	**<.001**	**-0.81**	-1.12, -0.51	-5.20	**<.001**
M×T	**1.29**	0.96, 1.62	7.72	**<.001**	**-1.19**	-1.64, -0.73	-5.12	**<.001**	**-0.69**	-1.00, -0.38	-4.42	**<.001**
P×Product	**-1.00**	-1.32, -0.67	-6.03	**<.001**	-0.17	-0.62, 0.28	-0.73	.47	-0.10	-0.40, 0.21	-0.62	.54
M×Product	**-0.79**	-1.11, -0.46	-4.74	**<.001**	-0.22	-0.67, 0.23	-0.96	.34	-0.19	-0.49, 0.11	-1.23	.22
T×Product	**-2.24**	-2.56, -1.91	-13.52	**<.001**	0.51	0.06, 0.96	2.21	**.03**	0.26	-0.05, 0.56	1.66	.10
P×M×T	**-1.25**	-1.71, -0.79	-5.29	**<.001**	1.05	0.41, 1.70	3.21	**.001**	**0.58**	0.14, 1.01	2.61	**.01**
P×M×Product	**0.55**	0.09, 1.01	2.34	**.02**	0.09	-0.55, 0.73	0.29	.78	0.05	-0.38, 0.48	0.23	.81

P×T×Product	**1.53**	1.07, 1.99	6.53	**<.001**	-0.16	-0.80, 0.48	-0.50	.62	-0.33	-0.76, 0.10	-1.51	.13
M×T×Product	**1.14**	0.68, 1.60	4.87	**<.001**	-0.03	-0.66, 0.61	-0.08	.94	0.02	-0.41, 0.45	0.07	.94
P×M×T×Product	**-0.67**	-1.32, -0.02	-2.01	**.04**	-0.05	-0.96, 0.85	-0.12	.91	0.03	-0.57, 0.64	0.11	.92

Random Effects

	Affect	*Concern*	*Support for regulation*
σ^2	1.04	2.00	0.91
τ_{00}	0.85 $_{ID}$	1.15 $_{ID}$	0.67 $_{ID}$
ICC	0.45	0.36	0.42
Marginal R^2 / Conditional R^2	0.49 / 0.72	0.21 / 0.50	0.19 / 0.53
